# Progression of lipase activity and pancreatic lipase immunoreactivity in dogs hospitalized for acute pancreatitis and correlation with clinical features

**DOI:** 10.1111/jvim.16591

**Published:** 2022-12-05

**Authors:** Claudia Cueni, Natalie Hofer‐Inteeworn, Claudia Kümmerle‐Fraune, Claudia Müller, Peter Hendrik Kook

**Affiliations:** ^1^ Clinic for Small Animal Internal Medicine Vetsuisse Faculty, University of Zurich Zurich Switzerland

**Keywords:** DGGR, dog, lipase activity, pancreatic ultrasonography, pancreatitis, PLI, Spec cPL

## Abstract

**Background:**

Lipase activity and pancreatic lipase immunoreactivity (PLI) have not been compared in dogs hospitalized for acute pancreatitis (AP).

**Objectives:**

To describe the progression of lipase activity and PLI, and correlations with clinicopathologic features in dogs with AP.

**Animals:**

Thirty‐nine dogs with AP based on clinical signs and lipase activity >350 U/L (reference interval [RI], 24‐108 U/L).

**Methods:**

Retrospective study. Lipase activity (LIPC Roche), PLI (SpecPL), and clinical signs were recorded daily. Admission (d1) data (clinical, laboratory, and ultrasound [US] findings), and clinical signs during hospitalization (d2‐d3) were assessed for correlation with lipases.

**Results:**

Median (range) duration of clinical signs before presentation was 2 days (1‐7 days). Median (range) lipase activity and PLI at d1 were 1070 U/L (range, 357‐1500 U/L) and 1111 μg/L (range, 292‐1500 μg/L). Strong correlation between assays at d1 (*r*_*s*_ 0.96; *P* < .0001; n = 39), remained equally strong on d2 (*r*_*s*_ 0.964; *P* < .0001; n = 39), and d3 (*r*_*s*_ 0.966; *P* < .0001; n = 22). On d2, lipase activity and PLI were within RI in 13/39 (33%) and 18/39 (46%) of cases. Lipase activities were minimally increased (median, 124 U/L) in 5 dogs with d2 PLI <200 μg/L. On d3, 4 more dogs had normal lipase activity and PLI, and the nature and magnitude of change were always the same for both assays. Clinical signs were not associated with lipases. Only a hyperechoic mesentery, but not an US diagnosis of AP, correlated significantly with lipase activity and PLI.

**Conclusions and Clinical Importance:**

Lipase decreases rapidly to near or within RI within 2 days of treatment in the majority of dogs with AP. Both lipase assays yielded virtually identical results. Mesenteric echogenicity may be an early marker of AP in dogs.

**Abbreviations**
AP: acute pancreatitis
DGGR: 1,2‐o‐dilauryl‐rac‐glycero‐3‐glutaric acid‐(6′‐methylresorufin) ester
IMHA: immune‐mediated hemolytic anemia
PLI: pancreatic lipase immunoreactivity
Spec cPL: specific canine pancreatic lipase
US: ultrasonography

## 1 INTRODUCTION

Acute pancreatitis (AP) is a common disorder in dogs. The diagnosis usually is based on a combination of clinical, laboratory, and imaging findings. In absence of histopathology, determination of serum lipase, either as a concentration (pancreatic lipase immunoreactivity [PLI]) or an activity (1,2‐o‐dilauryl‐rac‐glycero‐3‐glutaric acid‐(60‐methylresorufin) ester [DGGR]‐based lipase assays) is considered the laboratory test of choice.^1^, ^2^, ^3^, ^4^, ^5^, ^6^ Although both tests correlate highly,^2^, ^3^, ^5^, ^7^, ^8^, ^9^, ^10^, ^11^, ^12^ comparisons of both assays with clinical signs and routine laboratory evaluations performed at initial presentation are lacking. Comparison of lipase assays over time also has not been examined. Besides lipase measurement, pancreatic ultrasonography (US) is regarded the second cornerstone of a diagnosis of AP, although agreement and correlation of lipase activity and PLI concentration with pancreatic US have been repeatedly found to be low.^9^, ^11^, ^13^, ^14^, ^15^, ^16^ Emerging evidence suggests that duration of clinical signs before presentation affects lipase results and US evidence of pancreatitis differently.^11^ With shorter disease duration, dogs had significantly higher lipase activities but US was not positive significantly more often for pancreatitis.^11^ So far, the relationship between lipase and pancreatic US has not been assessed in dogs with clearly defined acute disease.

Therefore, we had the following objectives: First, to describe the time course of DGGR‐lipase activity and PLI and correlations with clinical signs in dogs with AP. Second, to determine if correlations exist between lipase activity and PLI and results of routine blood tests (CBC, serum biochemistry), as well as C‐reactive protein (CRP) concentration. Third, to determine if a correlation exists between both lipase assay results and US findings in a defined acute setting. We hypothesized that lipase activity and PLI concentration would decrease to results within or minimally above the reference interval (RI) within 1 to 2 days in the majority of dogs with AP and that results of baseline and follow‐up lipase activities and PLI concentrations would be interchangeable.

## 2 MATERIALS AND METHODS

### 2.1 Case selection and data collection

The study population of this retrospective study consisted of client‐owned dogs. All dogs were presented to the Clinic for Small Animal Internal Medicine of the Vetsuisse faculty, University of Zurich between January 2019 and August 2021. Inclusion criteria were hospitalization for AP, available consecutive measurements of lipase activity, available surplus serum for corresponding PLI determinations from the same samples that were used to measure lipase activity, and owner permission to use leftover samples. A suspicion (US evidence of pancreatic mass lesions) or confirmation of pancreatic carcinoma was an exclusion criterion. Diagnosis of AP was based on at least 1 of the following signs (lethargy, anorexia, vomiting, abdominal pain, diarrhea) of ≤7 day duration, and DGGR‐lipase activity >350 U/L (LIPC, Roche on Cobas, Roche Diagnostics, Rotkreuz, Switzerland using DGGR as substrate; RI, 24‐108 U/L).^9^, ^11^ This cutoff of 350 U/L is based on our clinical experience. At our institution, surplus blood samples routinely are stored at −30°C for 4 weeks. Once monthly, suitable samples were identified and sent out for PLI (Spec cPL) measurement (Diavet IDEXX, Switzerland). If sample volume allowed, previously unmeasured CRP concentrations also were measured. Similar to PLI (RI, 0‐200 μg/L) being reported up to 1500 μg/L, lipase activities were truncated at 1500 U/L for analyses. Recorded hematologic variables (Sysmex XN‐1000) consisted of hematocrit, platelets, segmented and band neutrophils, lymphocytes, neutrophil/lymphocyte ratio (NLR), and platelet/lymphocyte ratio (PLR). Leukocytes were recorded as concentration. In all cases, blood smears were manually evaluated for platelet clumping. Recorded serum biochemistry variables (Cobas c501, Roche Diagnostics, Basel, Switzerland) consisted of glucose, protein, albumin, alkaline phosphatase activity, alanine transaminase (ALT) activity, bilirubin, creatinine, urea, triglycerides, cholesterol, sodium, potassium, and chloride. Concentration of CRP was measured using an immunoturbidimetric assay (CANINE CRP, Randox laboratories).

All analyses were performed at the Clinical Laboratory of the Vetsuisse faculty, University of Zurich.

Blood sampling and abdominal US were performed within a median of 5 hours of each other (range, 1‐22 hours). Ultrasonography was performed either by a board‐certified radiologist or a resident of diagnostic imaging under direct supervision. Ultrasonography results were taken from radiology reports. Besides the final US diagnosis (pancreatitis yes/no), the following descriptive terms were recorded as present or absent for each patient: pancreatic enlargement, hypoechogenicity, mixed echogenicity, and hyperechoic mesentery. In all dogs, a standardized medical history was taken, and presenting clinical signs and physical examination findings were recorded. Fecal scores were assessed at admission and daily during hospitalization.^17^ During hospitalization the following clinical variables were recorded: general clinical demeanor (normal vs diminished), anorexia, vomiting, diarrhea, and abdominal pain.

### 2.2 Statistical analyses

Spearman's rank correlation coefficients (*r*_*s*_) between lipase activity and PLI, at baseline (d1) and days 2 to 3, as well as from all 3 days together were determined. Spearman's rank correlation coefficients also were used to assess correlations among lipase activity, PLI, CRP concentrations and US diagnosis (pancreatitis yes/no; dichotomized as binary variables [1/0 equivalent to presence/absence]), as well as the presence or absence of the 4 recorded US variables. Correlation coefficients were calculated between CBC and biochemistry variables mentioned above and lipase activity and PLI at d1. To address multiple comparisons of laboratory results with lipase activity and PLI, significant raw *P* values were adjusted using the *Q*‐value estimation for false discovery rate control.^18^ For significance of the *Q* value the 0.05 threshold was considered. Correlation coefficients also were used to assess correlations between clinical signs (general clinical demeanor [normal vs diminished], abdominal pain, inappetence, vomiting, hematemesis, and diarrhea) dichotomized as 1/0) and both lipase assay results at d1 to d3. Linear regression analysis was used to calculate the goodness‐of‐fit (*R* ^2^) of lipase activities with PLI results.

Mann‐Whitney *U* tests were used to compare lipase activity and PLI results between dogs with and without individual clinical signs and with and without individual US findings. A pairwise Wilcoxon test followed by a Bonferroni correction for multiple testing was used to compare lipase activity, PLI, and CRP concentrations between d1 and d3.

## 3 RESULTS

Hospitalization data of 39 visits met the inclusion criteria, with 1 dog presenting twice within 3 weeks with AP with hospitalization for 1 day each time, resulting in a total of n = 39 data sets. All following clinical, laboratory, and imaging data refer to n = 39 dogs. The median age was 8 years (range, 1‐14 years), there were 25 females (64%), of which 19 were spayed, and 14 males (36%), of which 10 were neutered. Most frequently represented were mixed‐breed dogs (5), Labrador Retriever (4), Bolonka zwetna (3), Miniature Schnauzer (3), Dachshund (2) Poodle (2), and Yorkshire Terrier (2).

### 3.1 Concurrent diseases

Concurrent diseases were: atopic dermatitis (1), cystitis (1), diabetes mellitus (1), early gallbladder mucocele (1), hypothyroidism (2), diet‐responsive protein‐losing enteropathy (1), mild chronic glomerular disease (1), and polyarthritis (1). One dog developed immune‐mediated hemolytic anemia (IMHA) on d2. One dog had received dexamethasone 1 day before presentation.

### 3.2 Treatment during hospitalization

All dogs received IV crystalloid fluids, antiemetics (maropitant, 38/39 [97%]; ondansetron, 17/39 [44%]; metoclopramide, 4/39 [10%]), and analgesics (metamizole, 28/39 [72%]; buprenorphine, 14/39 [36%]; methadone 10/39 (26%); paracetamol 2/39 [5%]). Antibiotics (ampicillin‐sulbactam) were given to 15/39 (38%) dogs. All dogs were offered a highly‐digestible low‐fat diet (Hill's Prescription Diet i/d Low Fat), or if refused, a highly‐digestible diet (Royal Canin Veterinary Diet Sensitivity Control or Essendia Exclusion Hypoallergenic Horse&Potato) as soon as vomiting had subsided. The diabetic dog received a constant rate infusion of insulin (NovoRapid, Novo Nordisk) and the dog that developed IMHA received prednisolone (2 mg/kg) on day 2.

### 3.3 Clinical signs at presentation

Median duration of clinical signs before presentation was 2 days (range, 1‐7). Fifteen dogs presented after 1, 14 dogs after 2, 5 dogs after 3, 2 dogs after 4 and 5 days, respectively, and 1 dog after 7 days. Clinical signs consisted of vomiting (33/39 [85%], in 9/33 [27%] dogs hematemesis), lethargy (29/39, 74%), anorexia (27/39, 69%), diarrhea (24/39, 62%), painful abdomen (13/39, 33%), polyuria and polydipsia (4/39, 10%), fever (2/39, 5%), and swollen joints (1/39, 3%). Median fecal score^17^ at d1 was 7 (range, 6‐7). Three dogs presented with only 1 clinical sign (2 with abdominal pain and 1 with vomiting).

The presence of clinical signs at d1 did not correlate with lipase activity or PLI (Table S1). Duration of clinical signs before presentation also did not correlate with lipase activity and PLI. Lipase activity and PLI were not significantly higher when recorded clinical signs were present or absent.

### 3.4 Clinical signs during hospitalization

On d2, a diminished general clinical demeanor was present in 19/39 (49%) dogs, diarrhea was present in 14/39 (36%) dogs (median fecal score of 7 [range, 6‐7]). Abdominal pain was present in 18/39 (46%), and 10/39 (26%) dogs were anorectic. Vomiting was present in 3/39 (8%) dogs. On d3, diminished general clinical demeanor was recorded in 6/22 (27%) dogs, diarrhea was present in 4/22 (18%) dogs with a fecal score of 6, abdominal pain was present in 9/22 (41%) dogs, and 2/22 (9%) dogs were anorectic. Vomiting was present in 2/22 (9%) dogs. Median duration of hospitalization was 3 days (range, 1‐10).

Neither lipase activity nor PLI concentration correlated significantly with the presence of individual clinical signs while hospitalized nor with duration of hospitalization (Table S1). Lipase activity and PLI were not significantly different between dogs when individual clinical signs were present or absent.

### 3.5 Time course of lipase activity and PLI concentrations

**FIGURE 1:**
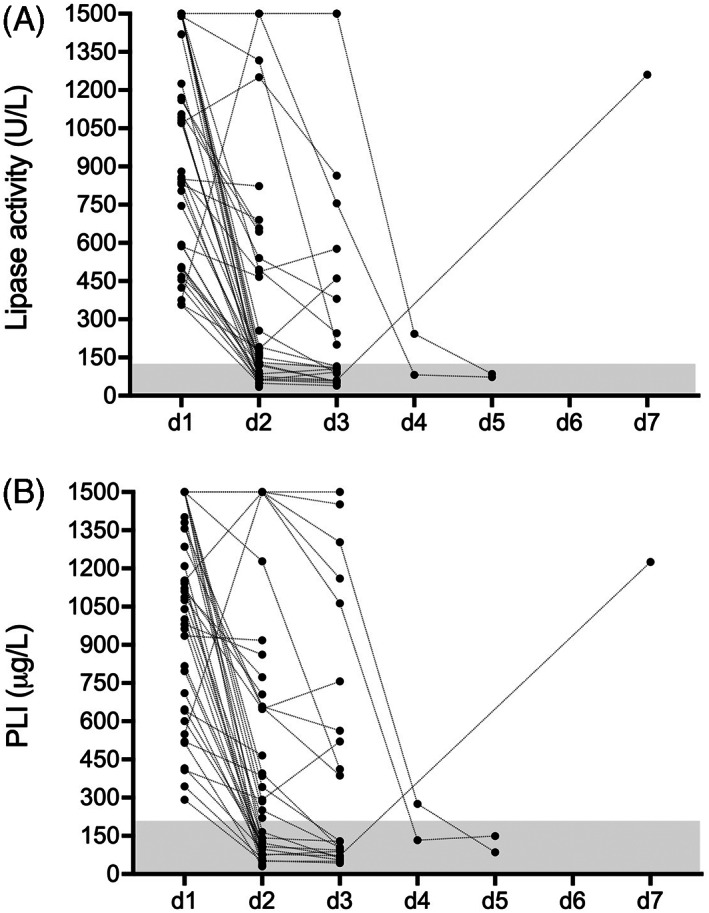
(A) Change in serum lipase activity over time in 39 hospitalized dogs with AP. The gray‐shaded area represents the RI. Please note that plotted points can be indistinguishable because of individual values that are too close or identical. (B) Change in serum PLI concentration over time in 39 hospitalized dogs with AP. The gray‐shaded area represents the RI. Please note that plotted points can be indistinguishable because of individual values that are too close or identical

The course of all lipase activities and PLI concentrations is shown in Figure 1A,B. At d1, median (range, interquartile range [IQR]) lipase activity and PLI concentration were 1070 U/L (357‐1500, 587‐1500) and 1111 μg/L (292‐1500, 647‐1402; Table 1). Correlation between both lipase assays at d1 was very strong (*r*_*s*_ 0.96; *P* ≤ .0001; n = 39) and remained equally strong on d2 (n = 39) and d3 (n = 22; Table 1). When all 100 paired lipase activity and PLI measurements (d1‐d3) were taken together, *r*_*s*_ was 0.981 (*P* < .0001).

**TABLE 1:** Spearman's rank correlation coefficient (*r*_*s*_ value) and statistical significance (*P* value) for the correlation between lipase activity and PLI concentration during hospitalization

Time	d1	d2	d3
n = dogs	39	39	22
*Lipase activity*			
Median (range, IQR)	1070 U/L (357‐1500, 587‐1500)	160 U/L (34‐1500, 77‐644)	113 U/L (39‐1500, 60‐592)
*PLI*			
Median (range, IQR)	1111 μg/L (292‐1500, 647‐1402)	250 μg/L (30‐1500, 98‐705)	129 μg/L (43‐1500, 72‐834)
*r*_*s*_ value	.96	.964	.966
*P* value	<.0001*	<.0001*	<.0001*

*Note*: An alpha level of 0.05 was used to determine statistical significance.

* Statistical significant value.

Linear regression analyses (100 paired measurements) indicated that lipase activity cutoffs corresponding to currently used PLI cutoffs of ≤200 and >400 μg/L were ≤151 and >348 U/L (*R* ^2^ = 0.96). The regression equation was: *y* = 0.987*x* − 46.91 (*y* = lipase activity and *x* = PLI). Standard error (SE) of the intercept was 18.53 and SE of the regression coefficient (*b*) was 0.02.

**FIGURE 2:**
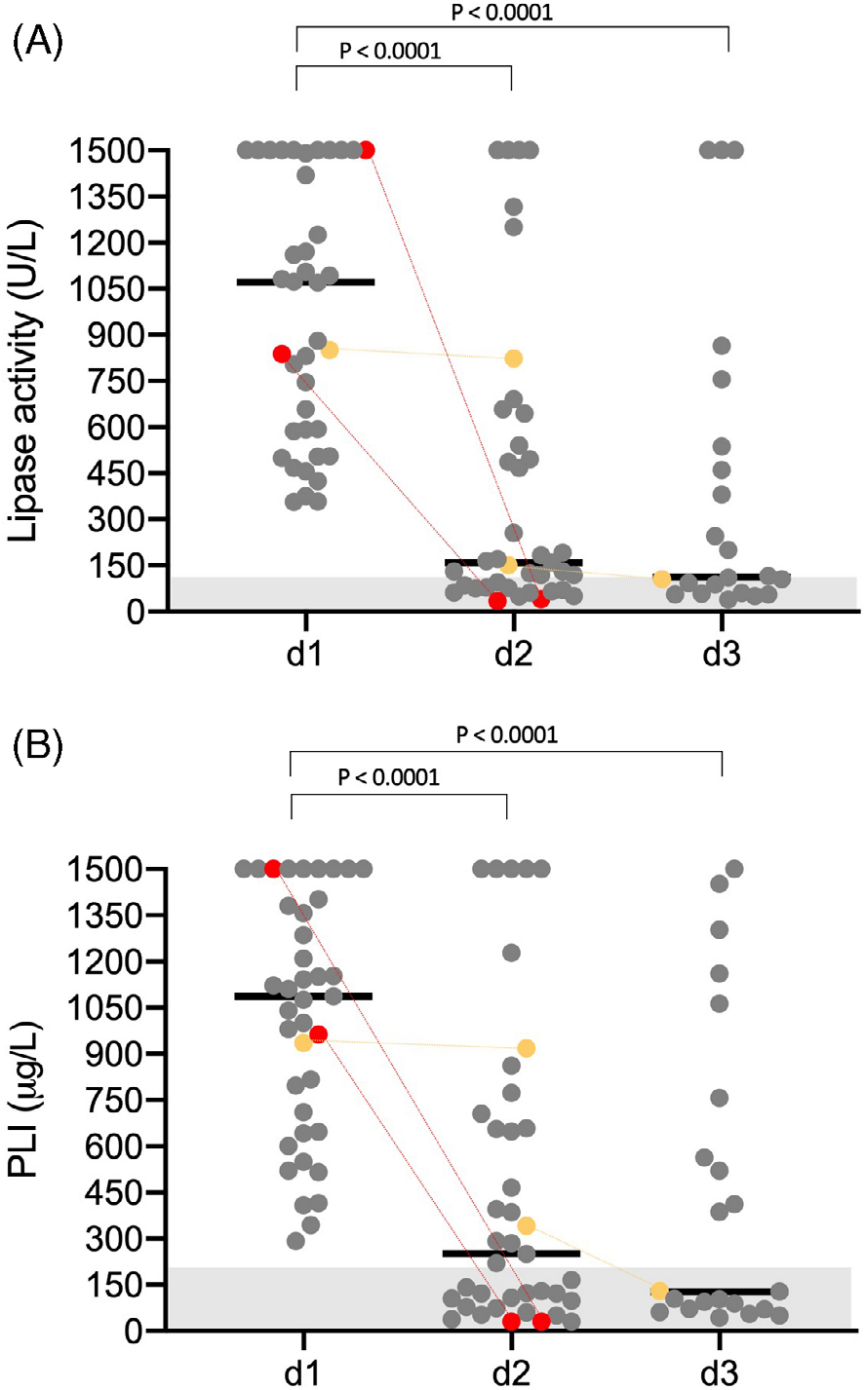
(A) Scatter plot of lipase activities measured during d1 to d3 in dogs with AP. Bars represent median values. Red circles symbolize values of one dog with two separate episodes of AP. Orange circles symbolize dogs treated with corticosteroids. Please note that plotted points can be indistinguishable because of individual values that are too close or identical. (B) Scatter plot of PLI concentrations measured during d1 to d3 in dogs with AP. Bars represent median values. Red circles symbolize values of one dog with two separate episodes of AP. Orange circles symbolize dogs treated with corticosteroids. Please note that plotted points can be indistinguishable because of individual values that are too close or identical

On d2, lipase activity and PLI had decreased significantly (*P* < .0001) compared with d1 (Figure 2A,B), but lipase activity and PLI results from d2 and d3 did not differ significantly (lipase activity, *P* = .47; PLI, *P* = .46).

On d2, 13/39 (33%) and 18/39 (46%) dogs had lipase activities and PLI within RI. Lipase activities of the five dogs with PLI concentration <200 μg/L on d2 were minimally increased (median 124 U/L; range, 119‐131). Three of these five dogs also had lipase activity and PLI measured on d3; results were within RI.

On d3, 2 dogs with persistently increased lipase activities (151 and 255 U/L) and PLI concentrations (342 and 396 μg/L) at d2, had results within RI (106 U/L, 108 U/L; 129 μg/L, 103 μg/L). Both dogs had lipase activity and PLI >1500 U/L and >1500 μg/L, respectively, at d1.

**FIGURE 3:**
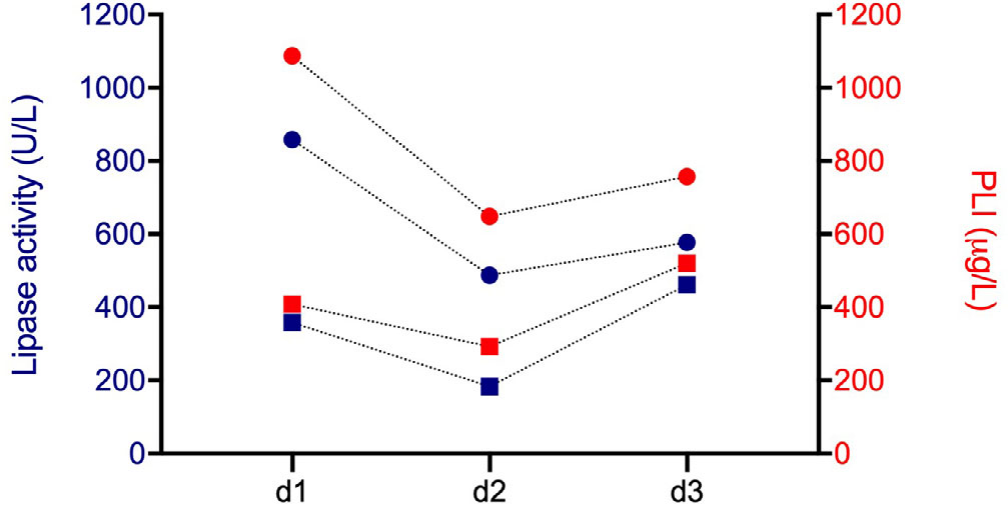
Lipase activity (blue) and PLI concentration (red) decreased at d2 and increased again at d3 in two dogs. Circles and squares symbolize lipase activity and PLI measurements from one patient, respectively

**FIGURE 4:**
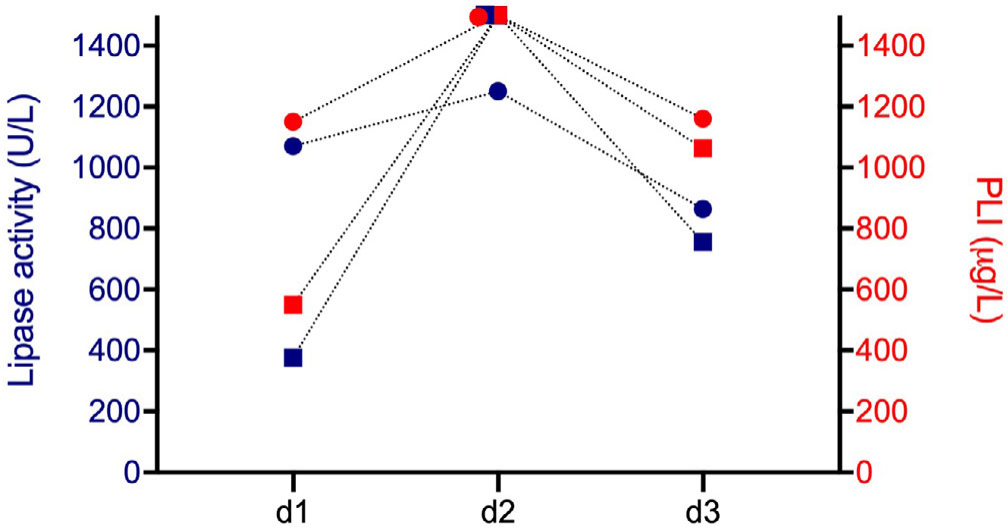
Lipase activity (blue) and PLI concentration (red) increased at d2 and decreased again at d3 in two dogs. Circles and squares symbolize lipase activity and PLI measurements from one patient, respectively

The nature and magnitude of change within the reported range of results were the same for both assays. In 2 dogs, lipase activity and PLI had decreased at d2 and increased again on d3 (Figure 3), whereas lipase activity/PLI had further increased at d2 and decreased again at d3 in another 2 dogs (Figure 4). In 3 dogs with lipase activity >1500 U/L and PLI >1500 μg/L at days 1 and 2, lipase activity remained >1500 U/L on d3 and PLI concentrations on d3 were 1500, 1452, and 1303 μg/L, respectively.

In 3 dogs, lipase measurements were available beyond d3. In two dogs with persistently increased lipase activity and PLI at d3, results further decreased to 243 U/L and 276 μg/L at d4 and were within RI at d5 in 1 dog, whereas both lipase results were within RI at d4 and d5 in the other dog (Figure 1A,B). In 1 dog with lipase activity and PLI at d2 and d3 within RI, lipase activity and PLI had returned to the initial level at d7 despite lack of relapsing clinical signs of AP (Figure 1A,B).

### 3.6 Correlation of lipase activity, PLI concentration, and laboratory variables

No hematologic variable correlated significantly with lipase activity or PLI. Only triglyceride concentration was moderately positively correlated with lipase activity (*r*_*s*_ 0.66; 95% CI: 0.369‐0.832; *P* ≤ .0001) and PLI (*r*_*s*_ 0.693; 95% CI: 0.421‐0.85; *P* < .0001). Both correlations remained significant after adjustment for false discovery rate control^18^ (both *Q* values = 0.0025). Activity of ALT was moderately positively correlated with lipase activity (*r*_*s*_ 0.453; 95% CI: 0.369‐0.832; *P* = .02). However, this finding was not significant after *P* value adjustment (*Q* value = 0.15). Triglyceride concentrations were within the RI (0.4‐1.5 mmol/L) in all (82%) but 5 dogs (median, 3.2 mmol/L; range 1.7‐48.8 mmol/L). No other biochemical variable was associated with lipase results.

### 3.7 CRP and correlations with lipase activity, PLI, and triglyceride concentrations

**FIGURE 5:**
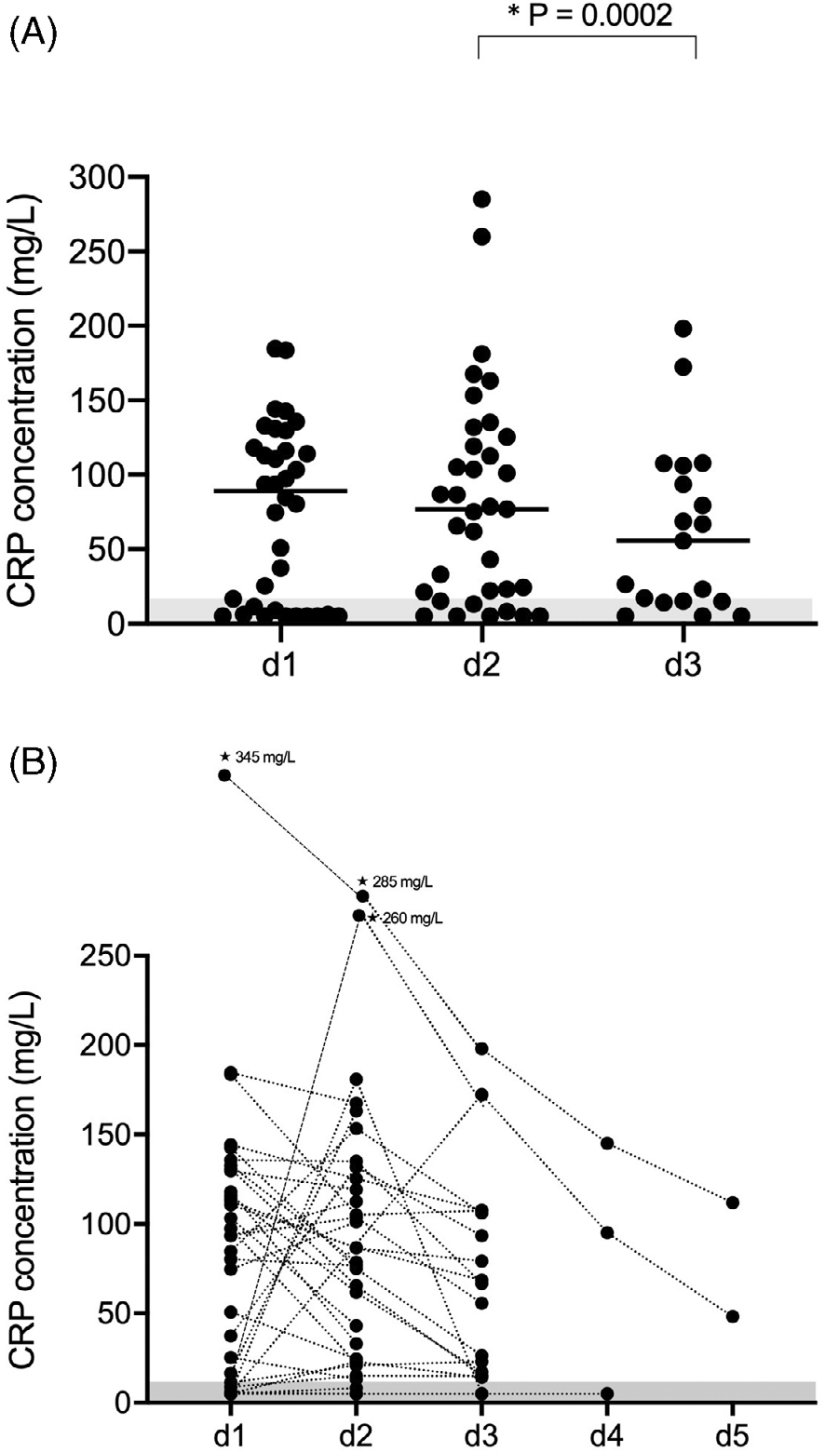
(A) Scatter plot of CRP concentrations measured at d1 to d3 in dogs hospitalized for AP. Bars represent median values. Values were compared with a paired Wilcoxon test followed by a Bonferroni correction. An alpha level of 0.0166 was used to determine statistical significance. CRP concentrations from d1 (median 98.1 μg/L, range 5‐345) were not significantly different from values at d2 (median, 76.8 μg/L, range 5‐285), while CRP concentration at d3 (median, 55.7 μg/L, range 5‐198) had significantly decreased compared with d2. (B) Changes in CRP concentrations over time in 36 dogs hospitalized with AP. The gray‐shaded area represents the RI

The CRP concentrations (median, IQR) at d1 (89 mg/L, 6.7‐126.8 mg/L) and d2 (76.8 mg/L, 21‐125.4 mg/L) were not significantly different (*P* = .36), but CRP concentration at d3 (91.3 μg/L, 14.9‐106.2 μg/L) had significantly decreased compared with d2 (*P* = .0002; Figure 5A). The CRP concentration increased from d1 to d2 in 13/36 (36%) dogs, and decreased from d1 to d2 in 20/35 (57%) dogs. At d3, CRP concentration had further decreased in 15/19 (79%) dogs, and minimally increased in 2 dogs. The CRP concentrations were normal (<10.7 mg/L) in 10/39 (26%) dogs at d1 and remained normal in 6/10 dogs (Figure 5B). A significant correlation of CRP with lipase (lipase activity, *r*_*s*_ = 0.321, *P* = .03; PLI, *r*_*s*_ = 0.373, *P* = .02) only was found on d2. No significant correlation was found when all 90 lipase activity and PLI and CRP pairs from d1 to d3 were combined and correlated. The CRP and triglyceride concentrations (n = 26) also did not correlate significantly.

### 3.8 Pancreatic US findings and lipase activity, PLI, and CRP concentration

Ultrasonography was performed in 35/39 (90%) dogs, and results were compatible with pancreatitis in 18/35 (51%) dogs, whereas the pancreas had a normal US appearance in 17/35 (49%) dogs. Neither lipase activity nor PLI correlated significantly with an US diagnosis of pancreatitis (Table 2). A significant correlation only was found between a hyperechoic mesentery and lipase activity and PLI concentration (Table 2). Lipase activity and PLI both also were significantly higher (1419 vs 722 U/L; *P* = .02, and 1285 vs 929 μg/L; *P* = .02) when a hyperechoic mesentery was present. Lipase activity and PLI were neither significantly higher in dogs with and without an US diagnosis of pancreatitis nor when a hypoechoic pancreas, mixed‐echoic, or enlarged pancreas was present.

**TABLE 2:** Spearman's rank correlation coefficient (*r*_*s*_ value) and statistical significance (*P* value) for the correlation between US findings and lipase activity, as well as PLI concentration

	US diagnosis of pancreatitis	Pancreatic hypoechogenicity	Pancreatic heterogenous echogenicity	Hyperechogenic mesentery	Pancreatic enlargement
*Lipase activity*					
*r*_*s*_ value	.192	.134	.117	.445	.246
*P* value	.28	.45	.51	.01*	.16
*PLI*					
*r*_*s*_ value	.269	.226	.123	.484	.341
*P* value	.12	.21	.49	.004*	.05

*Note*: An alpha level of 0.05 was used to determine statistical significance.

* Statistically significant value.

The CRP concentrations also did not correlate significantly with an US diagnosis of pancreatitis (n = 32 dogs). The CRP concentration was neither significantly higher in dogs with an US diagnosis of pancreatitis nor did CRP differ significantly between presence or absence of the four recorded US variables. One dog (7‐year‐old Shetland Sheepdog) presented twice within 3 weeks with hematemesis, anorexia, and apathy for 1 day and markedly increased lipase activities and PLI concentrations (marked red in Figure 2A,B). Upon first examination, the pancreas was of normal size, shape, and echogenicity. The mesentery was hyperechoic, and the US diagnosis was a hyperechoic mesentery in the cranial abdomen. During the second US examination, the pancreas was enlarged, with an irregular surface, rounded form, and mixed‐echoic parenchyma with a hyperechoic mesentery. The US diagnosis was AP. During both presentations, lipase activity and PLI completely decreased into RI within 24 hours (Figure 2A,B), and the dog was discharged at d2.

## 4 DISCUSSION

We described the course of disease and progression of lipase activity and PLI results in dogs hospitalized for AP. Lipase decreased rapidly to minimally increased activity or results were within RI in approximately half of the dogs within 1 day.

The strong correlation between both lipase assays at admission (d1) remained equally strong during the course of the disease. Whenever PLI decreased or increased, lipase activity decreased or increased and vice versa. The nature and magnitude of change were the same, and results of both assays were interchangeable from a clinical point of view. Minimal differences only were noticeable when considered in the context of the RI, but were clinically irrelevant. A concept of equivocal results is used for PLI interpretation. Results between 200 and 400 μg/L are regarded as equivocal, and results >400 μg/L as consistent with pancreatitis,^19^ but the basis for the 400 μg/L cutoff remains unknown. A recent review mentioned that PLI cutoffs were determined based on a case series of sick dogs with histopathologic evaluation of the pancreas.^20^ The only data matching this statement arise from a previous abstract.^21^ The PLI concentrations (measured using the original PLI ELISA) were increased above an “empirical cutoff value of 250 μg/L” in 9/11 dogs with histologically‐ confirmed pancreatitis.^21^ Dogs with pancreatitis had a median PLI concentration of 676.8 μg/L (no range reported).^21^ The empirical cutoff was not described in more detail, but based on the RI at the time, was 2.5× the upper RI. Form (acute vs chronic) and severity of pancreatitis was not reported.^21^ These early data refer to the original PLI assay developed at the Texas A&M Gastroenterology lab. The now commercially available PLI (Spec cPL) has been compared with the early PLI ELISA, and positive bias with approximately 50% higher concentrations for Spec cPL compared with the original PLI ELISA was found.^22^ This information means the above‐mentioned empirical 250 μg/L cutoff^21^ amounts to approximately 375 μg/L PLI (Spec cPL), which is close to 400 μg/L. In our study, a lipase activity cutoff of >348 U/L corresponded to a PLI of >400 μg/L in the regression analysis. Interestingly, this >348 U/L cutoff turned out to be approximately in the region of 3× the upper limit of the RI, a standard concept used to diagnose AP in humans and dogs.^8^, ^23^, ^24^ We had originally also created a preliminary equivocal range for the interpretation of lipase activity (109‐216 U/L^9^), but experience has taught us that the cutoff for a diagnosis of AP is somewhere between 300 and 350 U/L. Therefore we included only dogs with lipase activity >350 U/L in our study. However, we prefer to be more cautious with the use of rigid cutoffs because no peer‐reviewed information is available about how the 400 μg/L PLI cutoff was established, and it is virtually impossible to definitely diagnose or rule out milder forms of AP without highly invasive biopsies. Moreover, our results imply that disease duration before lipase measurement should be included in the interpretation of lipase in acute cases. For this reason, we refrain from wording such as “consistent with pancreatitis” and generally prefer “suspicious for pancreatitis.” It is precisely for these reasons that the concept of a 3× the upper limit of the RI is applied in human medicine. Lower diagnostic cutoffs have been described recently for DGGR‐based lipase activities.^5^, ^12^ Different assay methodology,^5^ different statistical approaches,^12^ and very likely inclusion of dogs with longer disease duration before the presentation may have played a role.

Comparative data evaluating lipase activity and PLI versus standardized histologic examination of the entire pancreas as reported in cats,^25^ are not available for dogs. Our findings illustrate that lipase activity results parallel PLI assay results that are widely regarded as highly specific for the diagnosis of pancreatitis. It has been claimed that DGGR‐based lipase activity assays are not specific for pancreatic lipase.^26^, ^27^ Recently, lipase activity was measured in six healthy dogs using a DGGR‐based assay (Diazyme Laboratories) at baseline and 10, 20, 30, 60, and 120 minutes after IV heparin administration.^27^ A significant difference (median difference of 4.3 U/L) was found between baseline and 10 minutes after IV heparin. This difference was most likely within RI, but no RI was given.^27^ The authors discussed that the substrate DGGR is not only hydrolyzed by pancreatic lipase but also by hepatic lipase and thus not pancreas‐specific.^27^ This is possible, but does not allow assessment of whether or not such a minimal difference has any clinical relevance.

No clinical sign correlated significantly with lipase activity, PLI, or CRP measurements, neither at admission nor during hospitalization. Lipase activity, PLI, and CRP concentration also were not significantly higher when clinical signs were present or absent. We were specifically interested in whether individual clinical signs rather than disease activity scores were associated with lipase activity or PLI. Scores may reflect disease severity but do not indicate the clinical problem. Because dogs usually are discharged when they have improved clinically and lipase results have been shown to decrease after treatment,^28^ it is not surprising to find an association between a clinical score and lipase result when all results from admission to discharge are taken together.^29^ A single study describes clinical signs in dogs with AP where duration of signs before the presentation was specified.^4^ In that study, 109 dogs had been sick for a median of 3 days (range, 0‐15 days). Inappetence or anorexia (94%) and abdominal pain (60%) were more common compared with our findings, but other clinical signs were approximately equally frequent.^4^ So far, lipase results have only once been compared with clinical signs and, in contrast to our results, lipase activity was significantly higher in dogs presented with abdominal pain, vomiting, hematemesis, and lethargy.^11^ Differences are likely related to study design. In the previous study,^11^ increased lipase results were not an inclusion criterion, and disease duration before presentation varied whereas all dogs in our study had an initial lipase activity >350 U/L and were acutely ill. Most probably, a larger group of dogs is needed to assess the relationship between clinical signs and lipase in AP. Disease duration may still be a crucial factor because initial lethargy or pain may not have been noticed by the owners. Studies with precise history‐taking are essential to examine a possible relationship between clinical and laboratory markers of pancreatitis. Nevertheless, lacking correlations between lipase activity and PLI and presenting clinical signs in the context of rapidly decreasing lipase activities and concentrations and the possibility of an ultrasonographically normal pancreas in early AP^16^, ^30^ suggest that each clinical sign must be considered equally.

Disease duration before presentation often is not described in dogs with pancreatitis. Most publications do not mention duration of clinical signs before the presentation.^1^, ^3^, ^5^, ^9^, ^12^, ^15^, ^16^, ^29^, ^30^, ^31^, ^32^, ^33^, ^34^, ^35^, ^36^, ^37^, ^38^, ^39^, ^40^ Some studies included dogs with a predefined disease duration, but did not report the actual duration of clinical signs.^6^, ^41^, ^42^ Some studies consider acute as short as 2 days,^43^ others consider 10 days,^6^ 2 weeks,^41^ or 3 weeks^44^ as acute. Lipase activities were significantly higher with shorter durations of clinical signs before presentation in a recent study,^11^ whereas the number of sick days before presentation did not correlate with lipase activity and PLI in our study. Delineating the time‐lipase relationship in the very acute phase of disease appears difficult, and it might be necessary to record the time factor in hours rather than days to detect a difference in the very acute process when considering the short half‐life of pancreatic lipase.^45^, ^46^

Two dogs had been treated with corticosteroids. Prednisolone can increase PLI concentrations into a diagnostic range for pancreatitis in healthy dogs,^47^ whereas effects on DGGR‐based lipase activity results are minimal and mostly within the RI.^48^ We purposely did not exclude these dogs, because we were curious if lipase activity and PLI would be affected differently. As can be seen from Figure 2A,B (corticosteroid‐treated dogs are marked orange), such was not the case.

Triglyceride concentration was the only laboratory variable significantly correlated with both lipase assays. We could not identify a correlation between CRP and triglyceride concentrations. This finding is in contrast to a recent study consisting of 31 dogs with AP where triglycerides were significantly correlated with CRP but not with PLI concentration at admission.^6^ This difference may have resulted from longer disease duration before presentation (up to 10 days) in the previous study^6^ when considering how rapidly lipase can decrease. Another reason might have been different study designs. Acute pancreatitis was diagnosed if PLI was ≥400 μg/L either on the day of presentation or the next day, but PLI results were not reported.^6^

The prevalence of hyperglycemia in our study was 37%, which was somewhat higher than the 30% reported in a previous study.^31^ Hyperglycemia also has been reported in dogs with experimentally‐induced AP.^49^ Data in humans suggest that hyperglucagonemia contributes to the hyperglycemia of AP.^50^ Other explanations are decreased glucose tolerance because of metabolic stress associated with systemic inflammation.^51^ We could not identify a correlation between glucose concentrations and lipase assays nor between glucose and CRP concentrations, and neither lipase activities nor PLI were higher when compared between dogs with normal and those with increased glucose concentrations. Stress hyperglycemia may have masked AP‐induced hyperglycemia in dogs and larger studies are needed to examine this relationship.

A recent study suggested that an increased NLR ratio provides useful information regarding the course of AP in dogs.^52^ In people, a recent meta‐analysis suggested an increased NLR has diagnostic value in predicting the severity of AP.^53^ In our study, NLR did not correlate significantly with lipase activity, PLI, or CRP at admission, and did not correlate with duration of hospitalization.

Systemic inflammation as assessed by the acute phase protein CRP did not correlate with lipase activity or PLI on d1, or when all measurements from days 1 to 3 were analyzed together, but the correlation was observed on d2. This finding is difficult to interpret given the predominant decrease of lipase results on d2 at the time of a mixed increase and decrease in CRP concentrations on d2. Pancreatic lipase release and systemic inflammation might not run in parallel, but a clearly defined onset of inflammation would be needed to better assess this observation. In contrast to our findings, PLI and CRP concentrations correlated significantly at first examination in 31 dogs with AP but not on the next day, but it was unclear on what day PLI was ≥400 μg/L.^6^ Recently, PLI and CRP were measured daily during hospitalization in 13 dogs with AP.^29^ No comparisons were made between days but, similar to our results, CRP increased and decreased during the first days of hospitalization.^29^ Other authors suggested CRP might be useful for monitoring recovery from AP at the fifth day after treatment.^32^ Concentration of CRP only was measured at admission and after 5 days, and not in between.^32^ Our results suggest that CRP is less useful in the first 2 days after admission. Perhaps no correlation was observed on d1 because CRP takes longer to peak, and no correlation may have been observed on d3 because the lipase peak was already over. Similarly, data in humans with AP indicate that CRP at admission is unpredictable of disease severity.^54^ The authors discuss that CRP is usually low in humans with AP if presentation is within a few hours of the onset of clinical signs because the hepatic synthesis of CRP peaks at 36 to 50 hours in people.^54^

We also were interested in how pancreatic US compared with lipase measurements in a clearly defined acute setting. Data in humans suggest that it takes ≥3 episodes of AP without morphological changes in the pancreas until morphological changes are detectable.^55^ Similarly, prevalence of imaging signs of AP in emergency department patients with lipase ≥3× upper RI is low in people.^56^ Also in dogs, US pancreatic changes consistent with AP may lag behind and occur later during hospitalization.^16^ Individual pancreatic US variables were not separately assessed in that study.^16^ We found that only a hyperechoic mesentery correlated significantly with lipase activity and PLI when dogs were acutely sick. Also, significantly higher lipase activity and PLI concentrations only were found when compared between dogs with and without a hyperechoic mesentery. Possibly, a hyperechoic mesentery represents an early marker for AP when the pancreas itself still appears unremarkable. Prospective studies are needed to verify this hypothesis.

Our study had some limitations. It would have been ideal to measure lipase activity, PLI, and CRP in all patients until all results decreased into the RI, but doing so was not possible in cases where clinical improvement preceded decreases in lipase. Also, in some cases there was not enough residual serum for CRP measurements. Documentation of a renewed sharp increase of lipase activity and PLI on d7 in one dog was only possible because the owner preferred to have the dog hospitalized longer than recommended and requested another laboratory assessment at discharge when the dog had no clinical signs. Furthermore, we could have included more cases without concurrent PLI measurement. However a goal of the study was to characterize the relationship between the 2 lipase assays. To better study the relationship of lipase and CRP, it would be ideal to include dogs with identical disease duration before presentation in future studies. Furthermore, all dogs in our study survived to discharge, thus it is possible that findings of our study are not necessarily applicable to more severe cases.

In conclusion, lipase activity and PLI can decrease rapidly in dogs with AP and reach near‐normal or normal results within 1 day in approximately 50% of cases even when markedly increased at first measurement. Thus, a diagnosis of AP can be missed if lipase determination is delayed by only 1 day. This observation is especially concerning when considering that pancreatic US can be normal in acute stages of pancreatitis. Mesenteric hyperechogenicity may be an early finding in AP when the pancreas itself is still normal. Results of the LIPC Roche and Spec PL assay were virtually the same and observed differences seemed clinically irrelevant. Those who advocate for PLI have repeatedly questioned the specificity of DGGR‐based lipase activity. Our results clarify this question for the LIP C Roche assay in dogs recovering from AP. Similar to previous results,^11^ a higher lipase activity cutoff (approximately 350 U/L) than what has been published earlier^9^ corresponds to the 400 μg/L PLI cutoff. However, cutoffs always should be interpreted in the context of the time course of the disease. Larger scale prospective studies are needed to address the factor of time before presentation and its relationship with laboratory as well as US evidence of AP in dogs.

## CONFLICT OF INTEREST DECLARATION

Authors declare no conflict of interest.

## OFF‐LABEL ANTIMICROBIAL DECLARATION

Authors declare no off‐label use of antimicrobials.

## INSTITUTIONAL ANIMAL CARE AND USE COMMITTEE (IACUC) OR OTHER APPROVAL DECLARATION

Authors declare no IACUC or other approval was needed.

## HUMAN ETHICS APPROVAL DECLARATION

Authors declare human ethics approval was not needed for this study.

## Supporting information


**Table S1.** Spearman's rank correlation coefficient (*r*_*s*_ value) and statistical significance (*P* value) for the correlation between lipase activity, PLI concentration, and the presence of clinical signs on d1. No significant correlations were found. An alpha level of 0.05 was used to determine statistical significance.
