# Supplementary material for: Progression of lipase activity and pancreatic lipase immunoreactivity in dogs hospitalized for acute pancreatitis and correlation with clinical features
Source: J Vet Intern Med. 2022 Dec 5;37(1):70–9. doi: 10.1111/jvim.16591 (PMC9889606; doi:10.1111/jvim.16591)
Supplement: Supplementary file 1 — Table S1. Spearman's rank correlation coefficient (r s value) and statistical significance (P value) for the correlation between lipase activity, PLI concentration, and the presence of clinical signs on d1. No significant correlations were found. An alpha level of 0.05 was used to determine statistical significance. [file JVIM-37-70-s001.pdf]

**Clinical signs on d1 in 39 dogs**	**n dogs (%)**	**Lipase activity**<br><br>**r_s value**<br>**P-value**	**PLI**<br><br>**r_s value**<br>**P-value**
Vomiting	33<br>(85)	-.09<br>.597	-.094<br>.586
Hematemesis	9<br>(23)	-.285<br>.087	-.286<br>.091
Lethargy	29<br>(74)	-.113<br>.506	-.115<br>.505
Anorexia	27<br>(69)	-.349<br>.198	-.319<br>.413
Diarrhea	24<br>(62)	-.216<br>.198	-.184<br>.283
Painful abdomen	13<br>(33)	.166<br>.328	.104<br>.548

Supplemental Table 1A denotes Spearman's rank correlation coefficient (r_s value) and statistical significance (P value) for the correlation between lipase activity, PLI concentration and the presence of clinical signs on d1. No significant correlations were found. An alpha level of .05 was used to determine statistical significance.

**Clinical signs on d2 in 39 dogs**	**n dogs (%)**	**Lipase activity**<br><br>**r_s value**<br>**P-value**	**PLI**<br><br>**r_s value**<br>**P-value**
Vomiting	3<br>(8)	-.06<br>.723	-.065<br>.702
Lethargy	19<br>(49)	-.125<br>.461	-.096<br>.574
Anorexia	10<br>(26)	.151<br>.372	.167<br>.323
Diarrhea	14<br>(36)	-.178<br>.293	-.141<br>.405
Painful abdomen	18<br>(46)	-.089<br>.602	-.107<br>.53

Supplemental Table 1B denotes Spearman's rank correlation coefficient (r_s value) and statistical significance (P value) for the correlation between lipase activity, PLI concentration and the presence of clinical signs on d2. No significant correlations were found. An alpha level of .05 was used to determine statistical significance.

Clinical signs on d3 in 22 dogs	n dogs (%)	Lipase activity<br><br>r_s value<br>P-value	PLI<br><br>r_s value<br>P-value
Vomiting	2 (9)	-.251<br>.3	-.251<br>.301
Lethargy	6 (27)	-.242<br>.318	-.275<br>.255
Anorexia	2 (5)	.243<br>.364	.232<br>.343
Diarrhea	4 (18)	-.142<br>.563	-.094<br>.7010
Painful abdomen	9 (41)	-.176<br>.472	-.234<br>.336

Supplemental Table 1C denotes Spearman's rank correlation coefficient (r_s value) and statistical significance (P value) for the correlation between lipase activity, PLI concentration and the presence of clinical signs on d3. No significant correlations were found. An alpha level of .05 was used to determine statistical significance.
